# PROTOCOL: Fear avoidance model psychological factors as predictors for persistent post‐concussion clinical outcomes: An integrative review

**DOI:** 10.1002/cl2.1311

**Published:** 2023-03-29

**Authors:** Katherine Buzzanca‐Fried, Jane Morgan‐Daniel, Aliyah Snyder, Russell Bauer, Sarah Lahey, Russell Addeo, Zachary Houck, Christopher Perez, Jason Beneciuk

**Affiliations:** ^1^ Department of Rehabilitation Science University of Florida Gainesville Florida USA; ^2^ University of Florida Health Science Center Libraries Gainesville Florida USA; ^3^ Department of Clinical and Health Psychology University of Florida Gainesville Florida USA; ^4^ Brooks Rehabilitation Department of Behavioral Medicine Jacksonville Florida USA

## Abstract

**Background:**

Persisting symptoms after concussion (PSaC) include physical, cognitive, and psychological symptoms which contribute to rehabilitation challenges. Previous research has not thoroughly investigated the association between PSaC and pain‐related psychological factors. Therefore, there is an opportunity to use current pain models, such as the Fear Avoidance Model (FAM), as a framework to explore these relationships. The goals of this integrative review are to (1) identify and describe range of evidence that explores relationships between psychological factors and clinical outcomes in patients with PSaC, and (2) develop a comprehensive understanding of FAM‐specific psychological factors that have been identified as potential predictors of clinical outcomes in patients with PSaC.

**Methods:**

This review will be based on principles and stages of an integrative review which will allow for inclusion of diverse methodologies: (1) problem formulation, (2) literature search, (3) data evaluation, (4) data analysis, and (5) presentation. Methods for reporting this review will be informed by the 2020 PRISMA guidelines for systematic reviews.

**Discussion:**

The findings from this integrative review will inform healthcare professionals working in post‐concussion rehabilitation settings regarding relationships between FAM psychological factors and PSaC—an area that until recently has not been thoroughly explored. Additionally, this review will inform the development of other reviews and clinical studies to further investigate relationships between FAM psychological factors and PSaC.

**Integrative Review Registration:**

OSF DOI 10.17605/OSF.IO/CNGPW.

## 1 BACKGROUND

Concussion, also known as mild traumatic brain injury, refers to a brain injury resulting in transient alteration of neurologic function and the presence of clinical signs and symptoms of injury (Kazl & Torres, 2019). Concussion is a significant public health problem, with nearly 30% of adults reporting sustaining a concussion in their lifetime (Daugherty et al., 2020). Although it is believed that the majority of patients with a concussion will recover by 3 months post‐injury (Levin & Diaz‐Arrastia, 2015; Wijenberg et al., 2017), a minority (1%–20% of the general population with concussion) will experience persisting symptoms after concussion (PSaC) (Bigler, 2008; Pundlik et al., 2020; Tator et al., 2016) and may require continued rehabilitation.

PSaC include reported physical (e.g., neck pain, headache, sleep disturbance), cognitive (e.g., attention, memory), and psychological/emotional (e.g., irritability, anxiety, etc.) symptoms which contribute to healthcare management challenges, particularly in rehabilitation settings (Rohling et al., 2012). For example, constant pain is a risk factor for developing long‐term psychological distress associated with catastrophic interpretations of pain, which reduces the likelihood of positive rehabilitation outcomes (Aaron et al., 2020). Inversely, pre‐injury psychological distress is a risk factor for PSaC, including those associated with pain/discomfort (e.g., headache, dizziness) (Bruffaerts et al., 2015), suggesting there is a reciprocal relationship between psychological distress and pain that serves to perpetuate symptoms and negatively influence patient outcomes.

Previous research has not thoroughly investigated the association between PSaC and pain‐related psychological factors. Thus, there is an opportunity to use current pain models as a framework to explore these relationships in this patient population. The Fear Avoidance Model (FAM) provides one relevant framework to describe the influence that specific psychological factors (e.g., pain catastrophizing, fear of pain) have on development and maintenance of chronic pain (Leeuw et al., 2007; Lethem et al., 1983). The FAM purports that behavioral avoidance leads to adverse physical and psychological consequences; however, recent studies suggest this may not necessarily be a linear process (Wideman et al., 2009, 2013). In addition, this cycle of pain experience, catastrophizing, and fear avoidant behaviors has been associated with the transition from acute to chronic pain (Hasenbring et al., 2014; Hruschak & Cochran, 2018). Although the FAM has primarily been applied to chronic pain (Crombez et al., 2012; Pincus et al., 2010; Wideman et al., 2009) it may also apply to those experiencing PSaC as fear‐avoidance has been reported for patients with PSaC (Silverberg et al., 2018; Wijenberg et al., 2017, 2020). As a result, application of the FAM may be relevant for this patient population through deconditioning, negative reinforcement, and extinction (Pittig & Wong, 2021). Based on the application of the FAM to chronic pain and its inclusion of psychological constructs relevant to concussion (i.e., anxiety, catastrophizing), the FAM will be used as the guiding framework for this review.

Current clinical practice guidelines for physical therapy and neuropsychology recommend psychosocial screening for post‐concussion patients (Marshall et al., 2015; Quatman‐Yates et al., 2020). As components of the FAM, anxiety and depression have been identified as predictors for concussion outcomes (Broshek et al., 2015; Iverson et al., 2017; Laliberté Durish et al., 2018; Losoi et al., 2016; Silverberg et al., 2018; Snell et al., 2020; Suhr & Spickard, 2012; Wijenberg et al., 2017, 2020), and assessment for these factors has been recommended in recent guidelines (Marshall et al., 2015; Quatman‐Yates et al., 2020). However, there is a current research gap with respect to the role other FAM‐specific psychological factors (e.g., catastrophizing, fear of pain, and fear avoidance) have on PSaC, therefore not surprising these factors are not recommended for screening in current clinical practice guidelines.

### 1.1 Protocol (aim/purpose)

The goals of this integrative review are to (1) identify and describe range of evidence that explores relationships between FAM‐specific related psychological factors (i.e., pain‐related fear, pain anxiety, negative affectivity, catastrophizing, hypervigilance, threat perception, fear avoidance, escape, disuse, depression, disability, confrontation, and recovery) and clinical outcomes in patients with PSaC, and (2) develop a comprehensive understanding of FAM specific psychological factors that have been identified as potential predictors of clinical outcomes in patients with PSaC.

#### 1.1.1 Research questions

Which FAM‐specific psychological factors predict the development of PSaC? Alternatively, which FAM‐specific psychological factors demonstrate relationships with PSaC clinical outcomes (i.e., domains) that may require further investigation as potential predictors of persistent post‐concussion clinical outcomes? Specific clinical outcome endpoints will be determined during the data extraction phase.

## 2 METHODS

This review will be based on principles and stages of an integrative review allowing for inclusion of diverse methodologies (i.e., experimental and non‐experimental study designs) and has potential to inform evidence‐based practice (Whittemore & Knafl, 2005). Five stages of integrative review include (1) problem formulation, (2) literature search, (3) data evaluation, (4) data analysis, and (5) presentation (Whittemore & Knafl, 2005). Methods for reporting this review will be further specifically informed by the 2020 PRISMA guidelines for systematic reviews (Page et al., 2021). As Toronto (2020, pp. 2–3) described, the critical differences between systematic and integrative reviews are that integrative reviews include theoretical and methodological literature to facilitate reviewing theories, models, and methodological issues. Integrative reviews may involve iterative development of the search strategy, screening questions, and other protocol elements; and do not typically include statistical synthesis of evidence. Previous studies have incorporated principles of an integrative review to guide a systematic review of the literature (Pringle et al., 2016).

### 2.1 Inclusion and exclusion criteria

Studies must be published in English and include human subjects who are 18 years of age and older that have been diagnosed with a concussion to be considered for inclusion. Non‐English publications will not be included in this study as the authors are limited to the English language and not able to properly analyze non‐English work. Additional inclusion criteria include studies that report: (1) FAM‐specific psychological factors associated with the development of PSaC or (2) FAM‐specific psychological factors associated with clinical outcomes for patients with persisting symptoms. Concepts must include FAM‐specific psychological factors concerning prognostic factors, predictors, associations, and relationships with post‐concussion outcomes. This integrative review will encompass empirical and theoretical publications and experimental (e.g., randomized clinical trial) and non‐experimental (e.g., cross‐sectional, cohort) study designs. Doctoral‐level dissertations are the only type of gray literature that will be considered for this review. There are no established publication date limits.

#### 2.1.1 Study designs

This integrative review is not limited to specific study designs and will encompass both empirical and theoretical publications and experimental (e.g., randomized clinical trial) and non‐experimental (e.g., cross‐sectional, cohort) study designs. Non‐standard study designs (i.e., Cluster‐randomized trials, cross‐over trials) will also be considered. Our rationale for this approach to inclusion based on study design is that we wanted to assure potentially relevant studies are not missed. Gray literature, with the exception of doctoral dissertations, will be excluded. Therefore, studies may be included regardless of publication status, such as non‐published doctoral dissertations. Once the authors have identified papers to be included, they will reach out to the authors of these publications and inquire about unpublished data that may be relevant to the review and that they are willing to share.

#### 2.1.2 Target population

To be included in the review, studies must involve adults (>18 years old) meeting one of the following clinical populations: post‐concussion, mild traumatic brain injury, concussion with post‐traumatic headache (PTH), or concussion with persistent pain. Studies containing non‐human subjects or participants under the age of 18 will be excluded. For the purpose of this review, we will focus on studies that diagnose concussion as a transient neurologic dysfunction resulting from a biomechanical force (Giza & Hovda, 2014). Studies that do not include subjects who meet the diagnoses criteria for concussion and that include cases of moderate to severe brain injuries, or brain injuries that result in abnormal imaging and detectable anatomic pathology, will be excluded. Studies in which only a subset of the sample is eligible for inclusion in the review will be included if separate analyses are conducted.

#### 2.1.3 Prognostic factors

Studies must include concepts related to FAM‐specific psychological factors as prognostic factors or predictors of PSaC. We will also include studies describing relationships or associations between psychological factors and post‐concussion clinical outcomes to further inform findings. Studies not including concepts related to psychological factors as prognostic factors or predictors of post‐concussion clinical outcomes will be excluded.

#### 2.1.4 Outcomes

Only studies reporting on clinical outcomes relating to PSaC will be considered. PSaC clinical outcomes include return to work, return to sport, cognition, emotion, quality of life, sleep, pain, and disability. These clinical outcomes are not used as a criterion for inclusion, and rather serve as a list of outcome domains of interest, therefore other outcomes may be included in final data extraction. Studies that do not include PSaC clinical outcomes will be excluded. Authors will determine if outcomes are clinical using the following operational definition: a clinical outcome is a change in symptoms, overall health, ability to function, quality of life, or survival outcomes that result from giving care to patients (NCATS, 2017). We did not select primary or secondary outcomes for this review as our aim was to gain greater comprehensive understanding of the relationship between FAM‐specific psychological factors and post‐concussion clinical outcomes. This approach is consistent with the purpose of integrative review methods. We do plan to report outcomes by clinical outcomes domain if able (e.g., pain, function).

### 2.2 Data management

Zotero (George Mason University), Covidence (Veritas Health Innovation Ltd.), and Excel (Microsoft Corporation) will be used to manage literature searches, screening, quality assessment, and data extraction across studies.

### 2.3 Literature search

#### 2.3.1 Search strategy

A finalized search strategy will be developed by a health sciences librarian and peer‐reviewed by a team of five collaborating neuropsychologists and experts in concussion. A preliminary test search in PubMed on March 11, 2021, generated approximately 1400 results. A search for existing related reviews or protocols was conducted on March 1, 2021, using the keywords (concussion OR post‐concussion OR “mild traumatic”) AND (predictor OR predictors). No directly relevant results specifically involving psychological factors were found in Biomed Central Systematic Reviews, Cochrane Database of Systematic Reviews, JBI Evidence Synthesis, JBI Systematic Review Register, or PROSPERO. Restrictions on search strategy including restrictions on publication date, format, or language will not be applied. The final search strategy will include medical subject headings (MeSH) terms that will be reported and go beyond the terminology used for our preliminary search.

#### 2.3.2 Electronic searches

Electronic information sources to be searched include the following databases: Biological Science Collection (ProQuest), CINAHL (EBSCOHost), EMBASE (Elsevier), PsycINFO (EBSCOHost), PubMed, SportDiscus (EBSCOHost), and Web of Science. Notably, PsycINFO and SportDiscuss were chosen as they are relevant to the concussion population. There are no specific trial registers (i.e., ClinicalTrials.gov, metaREGISTER) included in the search strategy. Additionally, there are no plans to search additional gray literature‐specific sources, as only doctoral dissertations will be considered for inclusion and most of the bibliographic databases that will be searched encompass gray literature.

#### 2.3.3 Searching other resources

Backward and forward citation searching will be conducted for all studies that advance to the data extraction stage of the review. Authors will conduct the forward reference and author searching by reviewing articles that have cited the original article, as well as reviewing the author's later or more current works to examine new developments. Further, if previous reviews on the same topic are identified, we will include the results of those review studies.

### 2.4 Data evaluation

#### 2.4.1 Study selection

All references identified will be downloaded from databases or journals and entered into Zotero (George Mason University, Fairfax, VA) with duplicates removed. Before full title and abstract screening, two reviewers will pilot test the screening of approximately 30 titles and abstracts to assess for preliminary consensus agreement. Title and abstract screening questions will include the following with a “yes” response required to be considered for inclusion:

1. Is this study in the English language?
2. Does this study contain only participants who are aged 18 years and older?
3. Does this study contain human participants?
4. Does this study report PSaC AND in relation to psychological factors? (Must include both PSaC and psychological construct to pass screening)

We will use Covidence, a systematic review screening tool, to organize the selection process. First, two reviewers will independently screen each title and abstract in reference and studies will be excluded by agreement. Any disagreement will be resolved through discussion or referral to a third reviewer if necessary. Second, included studies will undergo additional full‐text screening to confirm appropriateness for inclusion or provide rationale for exclusion at this stage if necessary, again using two reviewers for voting. Any disagreement will be resolved through discussion or referral to a third reviewer if necessary.

#### 2.4.2 Data extraction

Two independent reviewers will independently extract data from included studies using a standardized data extraction form in Excel designed for this review that will be piloted and refined before full data extraction. The original data collection form is included as a Supplemental File and data items for extraction include:

1. Study identifiers (authors, title, date published, journal, page numbers)
2. Study aims or objective
3. Study design
4. Study attrition (loss to follow‐up)
5. Clinical setting
6. Psychological factors measured (with specific measurement tools)
7. Are psychological FAM specific psychological factors measured? The Leeuw 2007, Pincus 2010, Crombez 2012, and Vlaeyen 2016, versions of the FAM will be referenced to determine relevance (Crombez et al., 2012; Leeuw et al., 2007; Pincus et al., 2010; Vlaeyen et al., 2016).
8. Clinical outcomes measured (with specific measurement tools)
9. Measure of association (adjusted and unadjusted measures of association (e.g., correlation coefficients, odds ratios, regression coefficients) will be extracted if provided. Effect sizes will be extracted to provide magnitude of relationship between psychological factor and clinical outcome. If not provided, effect sizes and standard errors will be calculated if able based on data provided, and any necessary data conversions will be described and reported. Any missing data will be reported)
10. Specific model, theory or framework in which study is grounded (if applicable)
11. Authors’ conclusions
12. Any pre‐planned data assumptions

#### 2.4.3 Missing data

Data missing will be reported and studies that contain missing data will still be considered for this review. If more than 50% of the above data extraction items are missing, the study will be excluded from this review. Authors will be contacted for any missing information to assist with data synthesis.

#### 2.4.4 Addressing multi‐arm studies

This integrative review is focused on predictive capabilities of psychological factors on post‐concussion outcomes and is not focused on intervention. However, intervention studies may be identified, among other study designs, and in the case that a study has more than two intervention arms, only intervention and control groups that meet eligibility criteria will be included. Multi‐arm studies will be analyzed in a way that avoids omission of relevant groups and the double counting of participants, such as the use of forest plots and multiple treatments meta‐analyses.

#### 2.4.5 Study grouping

We will group studies based on outcome domain assessed (e.g., return to work, return to sport, cognition, emotion, quality of life, sleep, pain, and disability) and study design to guide data synthesis in later stage. Different types of studies will be classified into major groups and then divided into subgroups as necessary and relevant (Pringle et al., 2016). This approach will allow to explore sources of heterogeneity to identify the impact of different psychological factors, outcome, follow‐up length, and study design.

#### 2.4.6 Quality appraisal

Two reviewers will independently assess the risk of bias for each outcome domain with a modified form of the Grading of Recommendations Assessment, Development and Evaluation (GRADE) tool (Guyatt et al., 2011) that has been used for previous prognostic factor reviews (Hayden et al., 2019; Huguet et al., 2013). GRADE will be used to rate the overall strength of evidence as “high,” “moderate,” “low” or “very low,” considering risk of bias (i.e., study participation, study attrition, prognostic factor measurement, outcome measurement, study confounding, and statistical analysis and reporting) (Hayden et al., 2019), imprecision, inconsistency, indirectness, publication bias, and size of observed effect. Any disagreement on risk of bias will be resolved through discussion or referral to a third reviewer. Before this stage of the review, two reviewers will pilot test unique items of the GRADE tool using a standardized checklist in conjunction with the Excel data extraction form.

### 2.5 Data synthesis

Data will be synthesized using narrative synthesis methods, relying on the use of words and texts to summarize and explain the findings of the synthesis (Popay et al., 2006). All extracted data points described above will be organized into an Excel spreadsheet. We anticipate all analysis will occur within and between study groupings (as described above) with potential to compare:

- FAM‐specific psychological factors
- Additional psychological factors relevant to the FAM (e.g., self‐efficacy)
- Outcome domains relevant to post‐concussion persistent symptoms
- Interactions with other relevant clinical factors (e.g., medical comorbidities)
- Specific models, theories, or frameworks

## 3 DISCUSSION

The overall purpose of this review is to identify literature on relationships between FAM‐specific psychological constructs (pain severity, pain catastrophizing, attention to pain, escape/avoidance behavior, disability, disuse, and vulnerabilities) (Leeuw et al., 2007) and PSaC and clinical outcomes. We acknowledge other potentially relevant psychological constructs (e.g., self‐efficacy) may be identified through this review, and those findings will be used to inform future research in this area. The background for this protocol indicates previous research is beginning to consider the relationships between FAM‐specific psychological factors and PSaC, however this area of research is yet to be integrated into a thorough review for this patient population. The methods outline the process of conducting an integrative review, including study selection, search methods, data extraction, and synthesis. Integrative reviews have the strength of considering theoretical and non‐theoretical literature and contributing to a theoretical framework by exploring successfully used methods. Thus, we aim to generate a comprehensive review and contribute to the current theory of the FAM for PSaC. The findings from this integrative review will inform healthcare professionals working in post‐concussion rehabilitation settings regarding relationships between FAM psychological factors and post‐concussion symptoms—an area that until recently has not been thoroughly explored. Additionally, this review will inform the development of other reviews and clinical studies to further investigate relationships between psychological factors and PSaC.

## 4 LIMITATIONS

Potential limitations of this integrative review include publication bias, as the only gray literature included is doctoral dissertations. Language bias is also a limitation as non‐English literature is not included. Additionally, reviewers will exclude studies focused on participants less than 18 years of age. Due to these exclusion criteria, reviewers could miss literature about the relationships between psychological factors and PSaC. Additionally, quality appraisal results using the modified GRADE can be variable due to the subjectivity of different raters (Mustafa et al., 2013), presenting another potential limitation.

## 5 CONCLUSION

Previous research has not thoroughly investigated the relationship between PSaC, pain, and psychological factors specific to the FAM. Therefore, there is an opportunity to explore current pain frameworks to gain further insight into these relationships in this patient population. This integrative review will identify the relationships between previously understudied psychological factors specific to the FAM and PSaC. Based on the findings, a theoretical framework of the FAM related to post‐concussion symptoms will be proposed, future directions for research will be discussed, and healthcare professionals working in post‐concussion rehabilitation settings will be informed about the complexity of how psychological factors impact post‐concussion rehabilitation outcomes.

## AUTHOR CONTRIBUTIONS

1. Katherine Buzzanca‐Fried: Doctoral student in the Rehabilitation Science program at the University of Florida contributed to conception of the project, drafted and revised the manuscript for important intellectual content; and is accountable for all aspects of the work. kbuzzanca@ufl.edu
2. Jason Beneciuk: Research assistant professor at the University of Florida Department of Physical Therapy and PhD mentor to Miss Buzzanca contributed to the conception of the project and interpretation, revised the manuscript for important intellectual content, and gave final approval of the manuscript. beneciuk@PHHP.UFL.EDU
3. Jane Morgan‐Daniel: University of Florida Health Science Center Libraries, contributed to the conception of the project (i.e., advising on methodology and reporting guidelines, conducting search strategy development, selecting databases, carrying out the final literature searches), revised the manuscript for important intellectual content, and gave final approval of the manuscript. morgandanie.jane@ufl.edu
4. Aliyah Snyder: Clinical assistant professor at the University of Florida Department of Clinical and Health Psychology, supervisory committee member to Miss Buzzanca, contributed to the conception of the project, revised the manuscript for important intellectual content, and gave final approval of the manuscript. aliyahsnyderphd@gmail.com
5. Russell Bauer: Professor at the University of Florida Department of Clinical and Health Psychology, supervisory committee member to Miss Buzzanca, contributed to the conception of the project, revised the manuscript for important intellectual content, and gave final approval of the manuscript. rbauer@PHHP.UFL.EDU
6. Sarah Lahey: Manager of Psychological services at Brooks Rehabilitation Hospital (Jacksonville, Fl), supervisory committee member to Miss Buzzanca, contributed to the conception of the project, revised the manuscript for important intellectual content, and gave final approval of the manuscript. Sarah. Lahey@Brooksrehab.org
7. Russell Addeo: Director of Behavioral Medicine at Brooks Rehabilitation Hospital (Jacksonville, Fl), contributed to the conception of the project, revised the manuscript for important intellectual content, and gave final approval of the manuscript. Russell.Addeo@Brooksrehab.org
8. Zachary Houck: Clinical Neuropsychologist at Brooks Rehabilitation Hospital (Jacksonville, Fl), contributed to the conception of the project, revised the manuscript for important intellectual content, and gave final approval of the manuscript. Zachary.Houck@Brooksrehab.org
9. Christopher Perez: Physical Therapist at Brooks Rehabilitation Hospital (Jacksonville, Fl), contributed to the conception of the project, will contribute to screening of titles and abstracts and gave final approval of the manuscript. Christopher.Perez-DeCorcho@Brooksrehab.org

Authors took various steps to mitigate publication bias, including using a health science center librarian with a team of clinical advisors to create the search strategy and select databases, searching a large number of databases, using four reviewers to search each title and abstract, not including publication date limits, and following PRISMA reporting guidelines

## COMPETING INTERESTS

None.

## ETHICS APPROVAL AND CONSENT TO PARTICIPATE

Not Applicable.

## CONSENT FOR PUBLICATION

Not Applicable.

## Supporting information

Supporting information.

Supplementary Information

## Data Availability

Not Applicable.
