# Supplementary material for: PROTOCOL: Fear avoidance model psychological factors as predictors for persistent post‐concussion clinical outcomes: An integrative review
Source: Campbell Syst Rev. 2023 Mar 29;19(2):e1311. doi: 10.1002/cl2.1311 (PMC10052450; doi:10.1002/cl2.1311)
Supplement: Supplementary file 2 — Supplementary Information [file CL2-19-e1311-s001.docx]

**List of Abbreviations**

PSaC: Persistent symptoms after concussion

FAM: Fear Avoidance Model

PTH: Post-traumatic headache
